# IMPLEMENTING AN AREA AGENCY ON AGING COMMUNITY CARE TRANSITION INITIATIVE: FACILITATORS AND BARRIERS

**DOI:** 10.1093/geroni/igad104.3422

**Published:** 2023-12-21

**Authors:** Antoinette Coe, Hao-Ping Chiang, Brigid Rowell, Nathaniel Bergman

**Affiliations:** University of Michigan College of Pharmacy, Ann Arbor, Michigan, United States; University of Michigan College of Pharmacy, Ann Arbor, Michigan, United States; University of Michigan College of Pharmacy, Ann Arbor, Michigan, United States; Region VII Area Agency on Aging, Bay City, Michigan, United States

## Abstract

The Michigan Region VII Area Agency on Aging (AAA) implemented a Community Care Transition Initiative (CCTI) for rural hospitalized older adults at high risk for readmission. After discharge home, AAA CCTI participants received a home visit by an AAA-trained pharmacy technician community health worker (CHW), a telehealth AAA pharmacist medication review, assessments by the CHW and pharmacist, education, primary care follow-up coaching, and referrals. As part of a program evaluation, we conducted six interviews with AAA key informants (e.g., pharmacist, director, CHWs). A semi-structured interview guide was piloted and revised to assess implementation, processes, impact, and future directions. Interviews were conducted via Zoom, recorded, transcribed, and checked for accuracy. Thematic analysis was used to identify implementation facilitators, barriers, and areas for program refinement. Three researchers coded the transcripts, developed a codebook, and resolved discrepancies by consensus. NVivo 14® was used for data management and analysis. Barriers to AAA CCTI implementation included unknowns at project start and need for procedures, anxiety about entering a client’s house, client’s lack of familiarity with technology, lack of perceived pharmacist benefit, limited personnel, and billing or financial reimbursement. Facilitators identified were the AAA’s reputation and backing, relationships built between healthcare providers, referral processes (e.g., community mental health), and pharmacist telehealth visit efficiency. Program refinements included shortened follow-up time and tailored visits to different client situations and needs. The results provide insights into the design of implementation strategies to disseminate the AAA CCTI to meet rural older adults’ health and social needs after hospitalization.

